# Improved Pre-attentive Processing With Occipital rTMS Treatment in Major Depressive Disorder Patients Revealed by MMN

**DOI:** 10.3389/fnhum.2021.648816

**Published:** 2021-06-08

**Authors:** Muzhen Guan, Xufeng Liu, Li Guo, Ruiguo Zhang, Qingrong Tan, Huaihai Wang, Huaning Wang

**Affiliations:** ^1^Department of Psychiatry, Xijing Hospital, Air Force Medical University, Xi’an, China; ^2^Department of Mental Health, Xi’an Medical University, Xi’an, China; ^3^School of Military Medical Psychology, Air Force Medical University, Xi’an, China; ^4^Department of Psychiatry, Xi’an No. 3 Hospital, the Affiliated Hospital of Northwest University, Xi’an, China; ^5^Department of Psychiatry, Xi’an Union Hospital, Xi’an, China

**Keywords:** major depressive disorder, repetitive transcranial magnetic stimulation, occipital lobe, mismatch negativity, event-related potentials

## Abstract

**Objectives:**

To investigate the improvement effect of occipital repetitive transcranial magnetic stimulation (rTMS) combined with escitalopram oxalate tablets on pre-attentive processing in patients with first-episode, medication-naive depression.

**Methods:**

Patients who were hospitalized between January and December 2019 were selected. They were randomly allocated to real occipital rTMS stimulation group with 27 cases receiving intermittent theta-burst (iTBS) and sham stimulation group with 24 cases over 20 days. The rTMS treatment target is located at the Oz point of the occipital region. Both groups took escitalopram oxalate tablets, and the average daily drug dose was 15.294 ± 5.041 mg. Hamilton Depression Rating Scale (HAMD) was used to assess the symptoms of depression before and after treatment, and mismatch negativity (MMN) was used to assess the improvement of pre-attentive processing before and after treatment.

**Results:**

After 20 days of treatment, the total score of HAMD (13.495 ± 3.700) in both groups was significantly lower than that before treatment [21.910 ± 3.841, *F*(1, 49) = 46, 3.690, *p* < 0.001]. After treatment, the latency of MMN in the real stimulation group (182.204 ± 31.878 ms) was significantly lower than that in the sham stimulation group (219.896 ± 42.634 ms, *p* < 0.001), and the amplitude of MMN in the real stimulation group (−7.107 ± 3.374 ms) was significantly higher than that in the sham stimulation group (−2.773 ± 3.7 32 ms, *p* < 0.001).

**Conclusion:**

Occipital rTMS treatment can enhance the early therapeutic effect and effectively improve the pre-attentive processing of patients with depression and provide a scientific basis for the new target of rTMS therapy in clinical patients with depression.

## Highlights

• Impairment of pre-attentive processing in major depressive disorder (MDD) is closely related to the abnormalities of the occipital lobe.
• Mismatch negativity (MMN) is an effective indicator for studying pre-attentive processing.
• Occipital repetitive transcranial magnetic stimulation (rTMS) can effectively improve pre-attentive processing in MDD.

## Introduction

Depression is a common mental illness characterized by persistent depressed mood and loss of interest (Suslow et al., 2020). Cognitive dysfunction is a common symptom of major depressive disorder (MDD), and it persists in the remission period. Cognitive dysfunction is a key factor in patients’ functional recovery (LeMoult and Gotlib, 2019). In recent years, more and more scholars have proposed “cognitive recovery” as a new target and new goal for the treatment of MDD (Bortolato et al., 2016). At present, neuroelectrophysiological and neuroimaging studies have found that the impairment of cognitive function in patients with depression is closely related to the structural and functional abnormalities of the occipital lobe (Shen et al., 2016; Zhang B. et al., 2016).

Recent research evidence shows that occipital cortex folding in patients with depression is abnormally increased, and it is highly positively correlated with the course and the severity of the disease, suggesting that occipital dysplasia may be a predictor of the course of depression (Schmitgen et al., 2019). In addition, functional magnetic resonance imaging (fMRI) and PET studies have found reduced occipital blood perfusion in patients with depression (Oda et al., 2003; Brockmann et al., 2009; Nagafusa et al., 2012; Qiu et al., 2014) and thickening of the occipital cortex (Ge et al., 2019) and high gray matter volume (Rubin-Falcone et al., 2018) in patients with first-episode, medication-naive depression. Voxel-based morphometric analysis has shown reduced occipital cortex volumes (Peng et al., 2016; Zhang H. et al., 2016). The structural alteration of the occipital lobe in patients with depression is also associated with occipital bending, which is an abnormality of neurodevelopment or anatomy (Maller et al., 2014). Fullard et al. (2019) proposed that occipital bending can be used as a structural biomarker for depression susceptibility and treatment effectiveness.

Occipital lobe function in patients with depression is also abnormal, and it is strongly associated with cognitive dysfunction. Task-related fMRI has shown that the reduction of occipital lobe activity in MDD patients has an impact on visual cognitive function, and the occipital lobe may be the initiating factor of cognitive function impairment (Li et al., 2018). Resting-state fMRI showed that the amplitude of low-frequency fluctuation (ALFF) in the left middle occipital gyrus was increased in MDD patients compared with healthy subjects, indicating that patients have hyperactivity in this brain region (Yu et al., 2017; Cheng et al., 2019), and it is positively correlated with the clinical symptoms of depression patients (Koshiyama et al., 2018; Taib et al., 2018). The results of resting-state functional connectivity showed that the intrinsic gray matter connectivity between the right lateral occipital cortex and the left temporal lobe and the right inferior lingual gyrus was attenuated in first-episode, medication-naive depression patients compared with healthy subjects (Zhang et al., 2019; Li et al., 2020).

Attention function runs through the entire cognitive process, including three contents: early pre-attentive processing, late alerting attention, and active attention (Koshiyama et al., 2018). Pre-attentive processing is a cognitive process that occurs before attention and is not dependent on consciousness, which reflects the unconscious and automatic processing of stimuli by the brain (Tivadar and Murray, 2019). Mismatch negativity (MMN) is an effective indicator for studying pre-attentive processing (Garrido et al., 2009). The research on pre-attentive processing in patients with depression is in its infancy. Studies have found that patients with depression have decreased MMN amplitude, impaired auditory and visual pre-attentive information processing, and decreased ability to detect changes in external information (He et al., 2010; Naismith et al., 2012; Qiao et al., 2013; Mu et al., 2016), which are closely related to clinical symptoms. Recent studies have found that memory comparison-based visual MMN for exploring external stimuli comes from the middle occipital gyrus (Maller et al., 2012; Kecskes-Kovdes et al., 2013). Li et al. (2020) found that the latency of MMN was prolonged and its amplitude was significantly reduced in patients with depression compared with healthy controls, indicating that patients with depression had a slowed speed of recognition of novel stimuli. Decreased MMN amplitude has become an important clinical indication for depression and other mental diseases (Näätänen et al., 2015).

The structural and functional damage of the occipital lobe in patients with depression is closely associated with cognitive impairment, especially with pre-attentive processing. As a region beyond the prefrontal cortex and its different subregions (Downar et al., 2014; Bakker et al., 2015; Fettes et al., 2018; Schulze et al., 2018), the occipital lobe may be a potential candidate target for the repetitive transcranial magnetic stimulation (rTMS) treatment of MDD. Zhang et al. (2020) demonstrated that left occipital rTMS provides effective and well-tolerated treatment, and fMRI measures functional change of the left occipital as a biomarker of therapeutic effect in MDD. Together, these results suggest that the occipital lobe may represent a highly promising target region for effective rTMS treatment in MDD (Zhang et al., 2020).

The improvement effect of drugs on the cognitive function of patients with depression is limited (Hartwigsen, 2016). Escitalopram oxalate tablets are widely used in clinical practice as a first-line antidepressant, but such drugs generally take effect in 2–4 weeks and cannot quickly control depression symptoms. As a noninvasive and safe neurophysiological technology, rTMS not only shows good prospects in regulating cognitive networks, promoting network remodeling, and improving cognitive dysfunction but also can accelerate or enhance the efficacy of antidepressant drugs (Cheng et al., 2018). It has been regarded as a treatment with great potential in the field of cognitive neuroscience that can promote cognitive function rehabilitation. Studies have found that stimulation of cortical areas in patients with depression using TMS can improve cognitive function, and this change may have a repair effect on the brain under pathological conditions (Verdon et al., 2004), which can well improve the cognitive function in depression, while there is no direct clinical evidence for the improvement of pre-attentive processing.

At present, there are few studies on pre-attentive processing in patients with depression, and there are even fewer studies on the improvement of pre-attentive processing by rTMS. Therefore, in this study, occipital lobe was used as a target for rTMS to investigate the improvement of pre-attentive processing in patients with depression before and after the treatment with occipital rTMS combined with escitalopram oxalate tablets, so as to provide clinical evidence for the new target of rTMS treatment for patients with depression.

## Subjects and Methods

### Participants

The enrolled patients were 51 first-episode, medication-naive patients with depression, who were hospitalized in the Department of Psychosomatics of the First Affiliated Hospital of Air Force Medical University between January and December 2019.

**Inclusion criteria:** (1) Meet the diagnostic criteria of MDD in the Diagnostic and Statistical Manual of Mental Disorders-Fifth Edition (DSM-V); (2) The 17-item score of Hamilton Depression Rating Scale (HAMD-17) is ≥ 18 points; (3) Meet the inclusion criteria and give informed consent. **Exclusion criteria:** (1) Have severe physical diseases; (2) Have a history of brain trauma or brain surgery; (3) The patient has a history of alcohol or substance abuse; (4) Have a history of other mental or neurological diseases. All subjects read and understood the experimental procedures and precautions before the experiment and signed an informed consent form. This study has been reviewed and approved by the Ethics Committee of the First Affiliated Hospital of Air Force Medical University (Approval document No.: KY20182047-F-1) and has been registered in the Chinese Clinical Trial Registry (Registration No.: ChiCTR1800019761).

### Method

#### Study Materials

In this study, the HAMD-17 (Lee et al., 2012) was used to assess the severity of depressive symptoms of the two groups of patients before and after rTMS treatment. The higher the score, the more severe the depression of the patients.

#### Treatment With rTMS

The repetitive transcranial magnetic stimulator used in this study is a magnetic field stimulator from YIRYIDE Medical (MagPro R30, Dantec Medtronic, Denmark, CCY-IA), and the stimulation coil is a 100-mm figure-of-eight-shaped coil. The resting motor threshold (RMT) is the minimum stimulation intensity that can elicit at least five motor evoked potential with an amplitude > 50 μV with 10 consecutive stimuli to the patient (Bashir et al., 2019). Intermittent theta-burst (iTBS) was delivered the intensity (120% RMT), differing only in stimulation pattern and total number of pulses (triplet 50-Hz bursts, repeated at 5 Hz; 2 s on and 8 s off; 600 pulses per session; total duration of 3 min 9 s). Initial treatment comprised 20 sessions in total (Blumberger et al., 2018). According to the international 10–20 system, the rTMS treatment target is located at the Oz point of the occipital region, and the stimulation coil is tangential to the scalp. The treatment location, intensity, frequency, and number of treatment of the sham stimulation group were the same as those of the real stimulation group, but the rTMS coil was perpendicular to the occipital region, and the head skin had the same “tapping” sensation, but the magnetic field did not enter the skull and had no stimulation effect. The rTMS treatment was performed twice a day for 20 consecutive days.

#### Mismatch Negativity Experimental Paradigm

The classical auditory Oddball test paradigm was used, the standard stimulus pure tone was set to 500 Hz and 80 dB, and the deviant stimulus was set to 2,000 Hz and 85 dB. The duration of the standard stimulus in each sequence was 50 ms, the duration of the deviant stimulus was 50 ms, and the ratio of standard stimulus to deviant stimulus was 8:2. The earphones were Hifiman RE-600, and the interstimulus interval (ISI) was 500 ms. The first 30 stimuli were standard stimuli, after which there were at least two standard stimuli before each deviant stimulus. The first 10 stimuli of the formal experiment were not processed.

#### Research Procedures

In this study, the antidepressant taken by the patients with depression was escitalopram oxalate tablets (brand: Lexapro), Xi’an Janssen Pharmaceutical Co., Ltd., National Medicine Standard J20150119, specification: 10 mg × 7 tablets. The drug was administered orally once daily. The usual dose was 10 mg per day, the maximum daily dose could be increased to 20 mg, and the average daily dose was 15.294 ± 5.041 mg. Patients in both groups used HAMD to assess the severity of depressive symptoms before and after the treatment with escitalopram oxalate combined with occipital rTMS and completed the MMN paradigm.

In the current study, we conducted a single-blind, randomized, sham-controlled study to test the acute efficacy and safety of the event-related potential (ERP)-based occipital rTMS in patients with MDD. The randomization program was created using a computer and executed by an investigator who is not involved in the treatment and recruitment of patients. The allocation of patients was screened, applying numbered in the sealed and opaque envelopes.

#### Electroencephalogram Recording

Electroencephalogram (EEG) was recorded using MEB-2306 electroencephalographic potentiometer manufactured by Nihon Kohden (Japan). The reference electrode was placed at the tip of the nose, and the ground electrode was placed at the forehead (Ground, FPz). At the same time, four electrode points of horizontal electrooculogram (HEOG) and vertical electrooculogram (VEOG) were used to record eye movements. The electrode impedance was less than 5 kΩ, the sampling rate was 500 Hz, and the band pass was 0.1–100 Hz.

EEG was recorded at the Fz and Cz electrode points from 100 ms before to 500 ms after stimulation according to the international 10–20 system. The baseline correction was performed at 100 ms before stimulation with a band pass of 0.1–20 Hz. Artifacts such as blinking, head movement, and electrode jitter were first eliminated by independent component analysis (ICA), and then the segments with amplitude greater than ± 75 μV were deleted. Those with an amplitude greater than ± 100 μV were considered as artifacts and were automatically eliminated during the superposition. MMN was obtained by subtracting the ERP of the standard stimulus from the deviant stimulus.

#### Statistical Analysis

The mean amplitudes of Fz and Cz electrode points were selected to perform repeated measures ANOVA, and the time window was 100–250 ms. Among them, stimulation type (two levels: standard stimulation and deviant stimulation), time (two levels: before and after treatment), and electrode position (two levels: Fz and Cz) were variables within the group, and groups (drug therapy combined with real rTMS stimulation group, drug therapy combined with sham rTMS stimulation group) were variables between groups. The statistical software used was SPSS 25.0. For data that did not conform to Mauchly “Spherical Symmetry” assumption of a normal distribution, the degree of freedom was corrected by the Greenhouse–Geisser method; *post hoc* pairwise comparisons were performed by the least significant difference (LSD) method, and the significance level was set to 0.05.

## Results

### Patient Demographics

They are Han Chinese and right-handed, including 21 males and 30 females, and they were 18–45 years old, with an average age of 30.118 ± 7.410 years. Their average education time was 11.863 ± 2.638 years, and their disease duration was 3.573 ± 2.857 months. There was no significant difference in demographic and clinical characteristics (Table 1).

**TABLE 1:** Demographic and clinical characteristics of the two groups.

	**Real stimulation (*n* = 27)**	**Sham stimulation (*n* = 24)**	***p***
Mean age (years)	31.111 ± 7.653	29.001 ± 7.120	0.52
Age range (years; min–max)	18–45	18–44	
Gender (female/male)	16/11	14/10	0.88
Education time (years)	12.233 ± 2.689	11.433 ± 2.531	0.36
Duration of current episode (months)	2.859 ± 2.130	4.375 ± 3.369	0.15

### Comparison of Hamilton Depression Rating Scale Total Scores Between the Two Groups Before and After Treatment

A 2 × 2 repeated measures analysis of variance was used to compare the total HAMD scores of the real stimulation group and the sham stimulation group before and after rTMS treatment. Among them, the main effect of time was significant [*F*(1, 49) = 463.690, *p* < 0.001, partial η^2^ = 0.904], and the total HAMD score after the treatment (13.495 ± 3.700) was significantly lower than that before the treatment (21.910 ± 3.841, *p* < 0.001). There was a time × group interaction, *F*(1, 49) = 5.986, *p* = 0.018, $\eta_{p}^{2}$ = 0.109. Further analysis showed that the total HAMD score of the real stimulation group and the sham stimulation group after treatment was significantly lower than the total HAMD score before treatment (*p* < 0.001), while there was no significant difference in the total HAMD score between the real stimulation group and the sham stimulation group after treatment (*p* > 0.05) (Table 2).

**TABLE 2:** Comparison of HAMD total scores before and after treatment between the two groups ($\bar{x}$ ± s).

**Group**	**Before treatment**	**After treatment**
Real stimulation (*n* = 27)	22.111 ± 4.098	12.741 ± 3.986***
Sham stimulation (*n* = 24)	21.708 ± 3.605	14.250 ± 3.247***

***p < 0.001. HAMD, Hamilton Depression Rating Scale.

### Event-Related Potential Results

**FIGURE 1:**
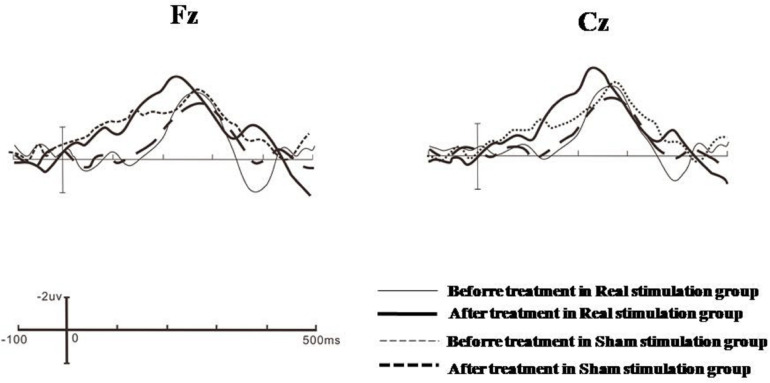
Grand averages of Fz and Cz electrode points in the real stimulation group and the sham stimulation group before and after treatment.

A 2 × 2 × 2 × 2 repeated measures analysis of variance was used to compare the changes of MMN latency before and after treatment in the drug combined with real rTMS stimulation group and the drug combined with sham rTMS stimulation group. The main effect of time was significant, *F*(1,49) = 47.302, *p* < 0.001, $\eta_{p}^{2}$ = 0.491, indicating that the latency after treatment (201.050 ± 37.256 ms) was significantly lower than that before treatment (223.006 ± 44.423 ms). There was a time × group interaction, *F*(1,49) = 30.907, *p* < 0.001, $\eta_{p}^{2}$ = 0.387. Further analysis showed that before treatment, there was no significant difference in MMN latency between the real stimulation group (221.907 ± 42.899 ms) and the sham stimulation group (224.104 ± 45.946 ms) (*p* = 0.860); after treatment, the MMN latency of the real stimulation group (182.204 ± 31.878 ms) was significantly lower than that of the sham stimulation group (219.896 ± 42.634 ms, *p* < 0.001) (see Table 3 and Figure 1 for details).

**TABLE 3:** Comparison of MMN latency between the two groups before and after treatment ($\bar{x}$ ± s).

	**Real stimulation group (*n* = 27)**		**Sham stimulation group (*n* = 24)**	
	**Before treatment**	**After treatment**	**Before treatment**	**After treatment**
Fz	221.778 ± 43.686	181.778 ± 32.765***	224.375 ± 46.122	220.333 ± 43.100
Cz	222.037 ± 42.113	182.630 ± 30.990***	223.833 ± 45.769	219.458 ± 42.167

***p < 0.001. MMN, mismatch negativity.

A 2 × 2 × 2 × 2 repeated measures analysis of variance was used to compare the changes of MMN amplitude before and after treatment in the drug combined with real rTMS stimulation group and the drug combined with sham rTMS stimulation group. The main effect of time was significant, *F*(1, 49) = 22.110, *p* < 0.001, $\eta_{p}^{2}$ = 0.311, indicating that the amplitude after treatment (−4.940 ± 4.136 μV) was significantly higher than that before the treatment (−3.842 ± 3.869 μV). The main effect of group was significant, *F*(1, 49) = 9.579, *p* = 0.003, $\eta_{p}^{2}$ = 0.164, indicating that the amplitude of the real stimulation group (−5.909 ± 3.391 μV) was significantly increased compared with that of the sham stimulation group (−2.874 ± 3.972 μV). There was a time × group interaction, *F*(1, 49) = 30.935, *p* < 0.001, $\eta_{p}^{2}$ = 0.388. Further analysis showed that before treatment, there was no significant difference in MMN amplitude between the real stimulation group (−4.711 ± 3.408 ms) and the sham stimulation group (−2.974 ± 4.213 ms) (*p* = 1.101); after treatment, the MMN amplitude of the real stimulation group (−7.107 ± 3.374 ms) was significantly higher than that of the sham stimulation group (−2.773 ± 3.732 ms, *p* < 0.001) (see Table 4 and Figure 1 for details).

**TABLE 4:** Comparison of MMN amplitude between the two groups before and after treatment ($\bar{x}$ ± s).

	**Real stimulation group (*n* = 27)**		**Sham stimulation group (*n* = 24)**	
	**Before treatment**	**After treatment**	**Before treatment**	**After treatment**
Fz	−4.781 ± 3.282	−7.387 ± 3.512***	−3.209 ± 4.127	−3.024 ± 3.863
Cz	−4.641 ± 3.534	−6.828 ± 3.237***	−2.739 ± 4.299	−2.523 ± 3.601

***p < 0.001. MMN, mismatch negativity.

## Discussion

The present study investigated the effect of occipital rTMS combined with pharmacotherapy on pre-attentional processes in patients with first-episode, drug-naive depression. The early efficacy of antidepressants has always been the focus of clinicians and patients. Studies have shown that early depressive symptom improvement will predict whether clinical recovery can be achieved in the future (Otte et al., 2016) and also affect the recovery of cognitive function. In this study, both the real stimulation group and the sham stimulation group took the first-line antidepressant escitalopram oxalate tablets. At the same time, the real stimulation group used the Oz point as the target of rTMS treatment for first-episode, medication-naive depression patients.

After 20 days of treatment with escitalopram oxalate tablets combined with occipital rTMS, the main finding from the study was that the depressive symptoms of the real stimulation group and the sham stimulation group were improved early, and their total HAMD scores were significantly decreased, which is similar to the results of previous studies (He et al., 2020). However, after treatment, there was no significant difference in the total HAMD score between the real stimulation group and the sham stimulation group. Although HAMD is used to evaluate the changes of depressive symptoms in clinical practice, in the evaluation process, subjective factors may lead to errors. Furthermore, the small number of subjects might be insufficient to show significant effects in HAMD scores. However, HAMD scores can well evaluate the effect on clinical symptoms in the two groups; the alteration of cognitive function is unknown. Therefore, in this study, we introduced electrophysiological indicators to further assess the cognitive function of patients with depression to provide an objective basis for early clinical judgment of the treatment effect.

Mismatch negativity is a sensitive electrophysiological indicator that reflects the pre-attention function. It is an ERP caused by novel stimulation. The difference wave obtained by subtracting the brain wave amplitude evoked by standard stimulation from the brain wave amplitude evoked by deviant stimulation is the MMN. The information can be processed automatically by investing no resources or less resources (May and Tiitinen, 2010). MMN usually appears in a time window of 100–250 ms after stimulation. Among the scalp electrodes, the MMN amplitude recorded by the frontal electrodes is the largest (Takegata et al., 2006).

Previous studies have shown that pre-attentive processing is impaired in patients with depression. Maekawa et al. (2012) found that the auditory MMN amplitude in patients with depression was smaller than the mean amplitude of the control group, suggesting that the pre-attentive processing of basic auditory information in patients with depression is impaired. Chang et al. (2010) used facial expression information to study pre-attentive processing in patients with depression and found that the vMMN amplitude was decreased in the early stage and disappeared in the late stage in patients with depression, indicating that the visual pre-attentive processing function of patients with depression is impaired. Importantly, to enhance the scientific basis and impartiality of assessment of the treatment effect, we conducted a sham-controlled randomized double-blind therapy study, in which the groups we compared included both real and sham structural MMN-based occipital rTMS targeting. In this study, we further analyzed the amplitude and latency of MMN and found that the MMN in the real stimulation group and the sham stimulation group had a long latency and a low amplitude, which is consistent with previous studies.

The main finding from the study was that occipital rTMS rapidly improved pre-attentive processing function with better efficacy. After 20 days of occipital rTMS treatment, the latency of MMN was shortened and the amplitude was increased in the real stimulation group compared with the sham stimulation group, indicating that after the TMS treatment of the occipital lobe, the patients were able to automatically process novel stimulation more quickly and devote more resources to pre-attentive processing, and their pre-attentive processing was improved (Hadas et al., 2019). Zhang et al. (2020) selected the visual cortex (V1) region of the left occipital lobe as the stimulation target of rTMS and found that after 5 days of rTMS treatment, the clinical symptoms of patients with depression were significantly improved, and the efficacy was maintained within 2 weeks. fMRI results showed that after occipital rTMS treatment, the cortical blood perfusion of patients with depression was increased, and task-state fMRI showed that the functional connection between the occipital lobe and the pre/subgenual anterior cingulated cortex was enhanced (Zhang et al., 2020). Therefore, occipital rTMS can improve the cognitive function of patients with depression (Speer et al., 2014).

Repetitive transcranial magnetic stimulation exerts antidepressant effects by giving magnetic stimulation to specific parts of the brain and has a good clinical effect (Bakker et al., 2015). In clinical practice, the selection of rTMS stimulation targets is directly related to the therapeutic effect of rTMS and has always been a research hotspot. In this study, occipital lobe was the target of rTMS, and the MMN components of electrophysiological therapy were selected to show that continuous occipital rTMS treatment can improve the pre-attentive processing of patients with depression, thereby improving the cognitive function of patients. Furthermore, our data indicated involvement of the occipital lobe, which may thus be used as a biomarker of therapeutic effect.

## Limitations and Conclusion

The present study has some limitations that should be taken into account. First, the sample size was relatively small, which might have resulted in inadequate statistical power to detect potential group differences in behavior. Although the small number of subjects might be sufficient to observe significant effects in the MMN study, a larger sample size might be necessary to clarify the group effects on behavioral performances (Yang and Ling, 2019). Moreover, the present study only compared the alterations of indicators before and after occipital rTMS combined with pharmacotherapy in treatment courses; however, it is unknown when the alterations happened. Future ERP studies provide a precise chronological delineation of alterations in the temporal dynamics underlying pre-attentive processing (Landes et al., 2018). Furthermore, we cannot investigate the neuroimaging mechanism of pre-attentive processing in MDD with occipital rTMS. However, future multimodal studies combining ERP and fMRI methods will allow to more comprehensively explore both the “when” and “where” of altered pre-attentive processing in MDD. Finally, depending on the geometry and orientation of the TMS coil, the magnetic field can still be sufficiently strong to result in effects (Duecker and Sack, 2015; Janssen et al., 2015); this could be the reason for no antidepressant effect difference between the groups.

Despite these limitations, this study is one of the first ERP studies to explore neurophysiological mechanisms of pre-attentive processing with occipital rTMS treatment in MDD. Our study investigated the effect of occipital rTMS combined with pharmacotherapy on pre-attentional processes in patients with first-episode, drug-naive depression. This study manifested that occipital rTMS treatment can effectively improve the pre-attentive processing in MDD patients. We encourage future work to explore predictive biomarkers for occipital rTMS treatment outcomes.

## Data Availability Statement

The raw data supporting the conclusions of this article will be made available by the authors, without undue reservation.

## Ethics Statement

The studies involving human participants were reviewed and approved by the Ethics Committee of the First Affiliated Hospital of Air Force Medical University. The patients/participants provided their written informed consent to participate in this study.

## Author Contributions

XL, MG, and QT conceived and designed the experiments. LG and RZ performed the experiments. MG and HHW analyzed the data. HNW and QT conceived the project and modified the manuscript. All authors read and approved the final manuscript.

## Conflict of Interest

The authors declare that the research was conducted in the absence of any commercial or financial relationships that could be construed as a potential conflict of interest.
